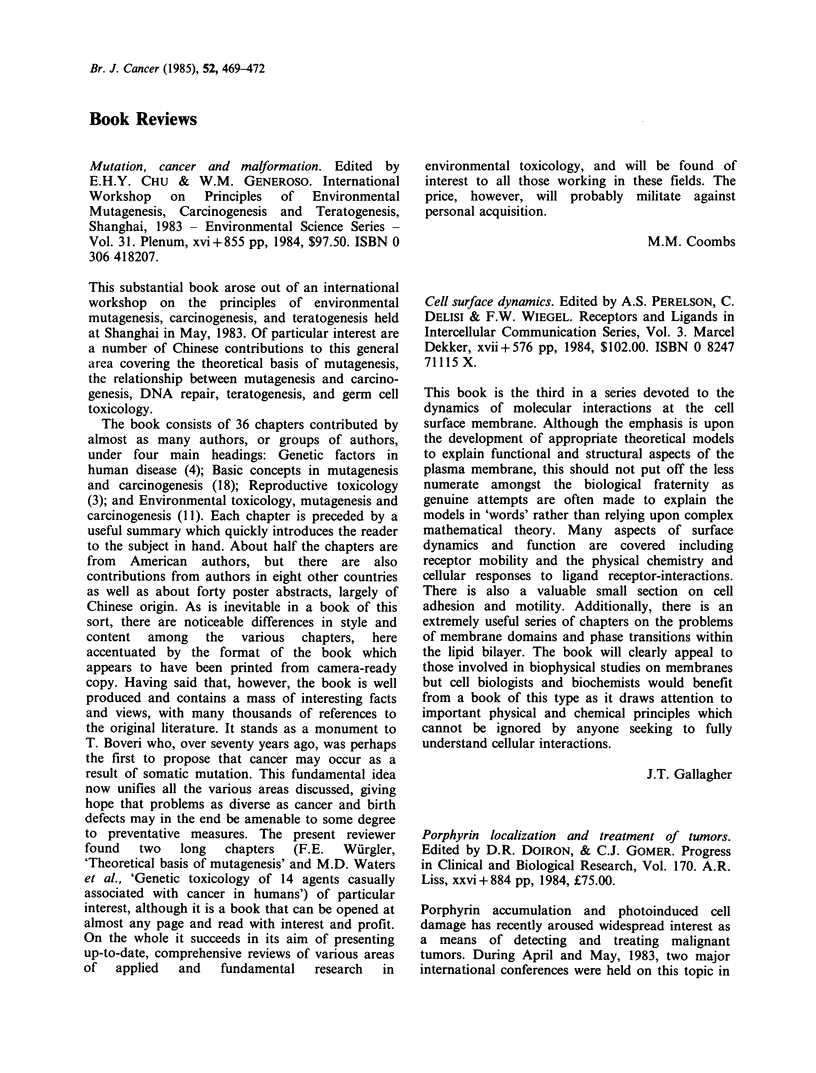# Mutation, cancer and malformation

**Published:** 1985-09

**Authors:** M.M. Coombs


Br. J. Cancer (1985), 52, 469–472

### Book Reviews

*Mutation, cancer and malformation.* Edited by E.H.Y. CHU & W.M. GENEROSO. International Workshop on Principles of Environmental Mutagenesis, Carcinogenesis and Teratogenesis, Shanghai, 1983 – Environmental Science Series – Vol. 31. Plenum, xvi+855 pp, 1984, $97.50. ISBN 0 306 418207.

This substantial book arose out of an international workshop on the principles of environmental mutagenesis, carcinogenesis, and teratogenesis held at Shanghai in May, 1983. Of particular interest are a number of Chinese contributions to this general area covering the theoretical basis of mutagenesis, the relationship between mutagenesis and carcinogenesis, DNA repair, teratogenesis, and germ cell toxicology.

The book consists of 36 chapters contributed by almost as many authors, or groups of authors, under four main headings: Genetic factors in human disease (4); Basic concepts in mutagenesis and carcinogenesis (18); Reproductive toxicology (3); and Environmental toxicology, mutagenesis and carcinogenesis (11). Each chapter is preceded by a useful summary which quickly introduces the reader to the subject in hand. About half the chapters are from American authors, but there are also contributions from authors in eight other countries as well as about forty poster abstracts, largely of Chinese origin. As is inevitable in a book of this sort, there are noticeable differences in style and content among the various chapters, here accentuated by the format of the book which appears to have been printed from camera-ready copy. Having said that, however, the book is well produced and contains a mass of interesting facts and views, with many thousands of references to the original literature. It stands as a monument to T. Boveri who, over seventy years ago, was perhaps the first to propose that cancer may occur as a result of somatic mutation. This fundamental idea now unifies all the various areas discussed, giving hope that problems as diverse as cancer and birth defects may in the end be amenable to some degree to preventative measures. The present reviewer found two long chapters (F.E. Würgler, 'Theoretical basis of mutagenesis' and M.D. Waters *et al.*, 'Genetic toxicology of 14 agents casually associated with cancer in humans') of particular interest, although it is a book that can be opened at almost any page and read with interest and profit. On the whole it succeeds in its aim of presenting up-to-date, comprehensive reviews of various areas of applied and fundamental research in environmental toxicology, and will be found of interest to all those working in these fields. The price, however, will probably militate against personal acquisition.

M.M. Coombs